# Functional Analysis of Novel Candidate Regulators of Insulin Secretion in the MIN6 Mouse Pancreatic β Cell Line

**DOI:** 10.1371/journal.pone.0151927

**Published:** 2016-03-17

**Authors:** Masaki Kobayashi, Eiji Yamato, Koji Tanabe, Fumi Tashiro, Satsuki Miyazaki, Jun-ichi Miyazaki

**Affiliations:** 1 Department of Stem Cell Regulation Research, Graduate School of Medicine, Osaka University, 2–2 Yamadaoka, Suita, Osaka, 565–0871, Japan; 2 Department of Nutrition and Food Sciences, Mukogawa Women’s University, 6–46 Ikebiraki-cho, Nishinomiya, Hyogo, 663–8558, Japan; Communaute d\'Universites et d\'Etablissements Lille Nord de France, FRANCE

## Abstract

Elucidating the regulation of glucose-stimulated insulin secretion (GSIS) in pancreatic β cells is important for understanding and treating diabetes. The pancreatic β cell line, MIN6, retains GSIS but gradually loses it in long-term culture. The MIN6 subclone, MIN6c4, exhibits well-regulated GSIS even after prolonged culture. We previously used DNA microarray analysis to compare gene expression in the parental MIN6 cells and MIN6c4 cells and identified several differentially regulated genes that may be involved in maintaining GSIS. Here we investigated the potential roles of six of these genes in GSIS: *Tmem59l* (Transmembrane protein 59 like), *Scgn* (Secretagogin), *Gucy2c* (Guanylate cyclase 2c), *Slc29a4* (Solute carrier family 29, member 4), *Cdhr1* (Cadherin-related family member 1), and *Celsr2* (Cadherin EGF LAG seven-pass G-type receptor 2). These genes were knocked down in MIN6c4 cells using lentivirus vectors expressing gene-specific short hairpin RNAs (shRNAs), and the effects of the knockdown on insulin expression and secretion were analyzed. Suppression of *Tmem59l*, *Scgn*, and *Gucy2c* expression resulted in significantly decreased glucose- and/or KCl-stimulated insulin secretion from MIN6c4 cells, while the suppression of *Slc29a4* expression resulted in increased insulin secretion. *Tmem59l* overexpression rescued the phenotype of the *Tmem59l* knockdown MIN6c4 cells, and immunostaining analysis indicated that the TMEM59L protein colocalized with insulin and GM130, a Golgi complex marker, in MIN6 cells. Collectively, our findings suggested that the proteins encoded by *Tmem59l*, *Scgn*, *Gucy2c*, and *Slc29a4* play important roles in regulating GSIS. Detailed studies of these proteins and their functions are expected to provide new insights into the molecular mechanisms involved in insulin secretion.

## Introduction

Glucose-stimulated insulin secretion (GSIS) from pancreatic β cells is essential for the regulation of blood glucose levels. Although GSIS in β cells has been intensively studied, the underlying mechanisms have not been fully elucidated. As reviewed in [1], the time course of GSIS displays a biphasic pattern. The first-phase insulin release begins soon after the glucose stimulation and persists only for a few min and is followed by the second phase, which lasts for 2–3 h. This biphasic pattern is observed *in vivo* and *in vitro*. In the pathogenesis of type 2 diabetes, the earliest detectable defect in β-cell function is generally thought to be a reduction in first-phase insulin secretion [2].

The MIN6 cell line, which was established from an insulinoma of a transgenic mouse expressing the SV40 T antigen in pancreatic β cells, secretes insulin in response to physiological stimuli and is a useful tool for studying the mechanisms of insulin secretion [3]. However, since MIN6 cells in long-term culture with repeated passages lose their ability to secrete insulin in response to glucose, we isolated a subclone (MIN6c4) that retains GSIS even after long-term culture [4]. To identify genes involved in maintaining GSIS, we previously compared the gene expression profiles of four groups of MIN6 cells: parental MIN6 cells at low passage (Pr-LP) and high passage numbers (Pr-HP), and MIN6c4 cells at low passage (C4-LP) and high passage numbers (C4-HP). From this analysis, we identified a group of genes whose expression was high in the glucose-responsive Pr-LP, C4-LP, and C4-HP cells, but was extremely low in the nonresponsive Pr-HP cells, as candidate genes that may be involved in the maintenance of GSIS [4]. Analysis of these genes and their protein products is expected to extend our understanding of the molecular mechanisms of GSIS.

Other groups also performed microarray-based analyses between low-passage and high-passage MIN6 cells and between well-regulated and dysregulated MIN6 subclones, and identified a number of differentially expressed genes, which included the genes related to secretory pathway, metabolism, cell adhesion, and so forth [5,6]. These studies showed that microarray-based approach provides a useful tool for identifying genes involved in GSIS of β cells.

Neuronal cells and pancreatic β cells express many common genes involved in the mechanism of secretion [7,8], and we speculated that these commonly expressed genes might play roles in the insulin secretion from β cells. Using the databases Unigene (http://www.ncbi.nlm.nih.gov/unigene/) and T1Dbase (http://www.t1dbase.org/page/AtlasHome), we selected six of the candidate genes identified in our previous study, whose expression was high in both cell types [*Tmem59l* (Transmembrane protein 59 like), *Scgn* (Secretagogin), *Gucy2c* (Guanylate cyclase 2c), *Slc29a4* (Solute carrier family 29, member 4), *Cdhr1* (Cadherin-related family member 1), and *Celsr2* (Cadherin EGF LAG seven-pass G-type receptor 2)] for further investigation. In the present study, we analyzed the effects of knockdown of these genes on GSIS in MIN6c4 cells.

## Materials and Methods

### MIN6c4 cell culture

MIN6c4 cells were maintained in Dulbecco’s modified Eagle’s medium (DMEM) supplemented with 25 mM glucose, 13% heat-inactivated fetal bovine serum, and 0.1 mM 2-mercaptoethanol in humidified 5% CO_2_ at 37°C, as described previously [4]. We used the MIN6c4 cells at passage 40–50.

### Quantitative RT-PCR analysis of RNA from MIN6 cells

Total RNA was extracted from MIN6 cells by the acid guanidinium-phenol-chloroform (AGPC) method and subjected to cDNA synthesis using ReverTra Ace α (Toyobo, Tokyo, Japan). Quantitative RT-PCR analysis was carried out using FastStart Universal SYBR Green Master (Rox) (Roche, Basel, Switzerland). The reaction was performed with a 7300 Real-Time PCR System (Applied Biosystems, Foster City, CA, USA) using the following thermal cycling conditions: 95°C for 10 s followed by 40 cycles of 95°C for 5 s and 60°C for 31 s. The relative expression levels of the target genes were normalized to that of *Rpl32* [4]. The sequences of the primers used are shown in Table 1.

**Table 1:** PCR primers used in the present study.

Gene	Forward (5' to 3')	Reverse (5' to 3')
***Tmem59l***	**CCAATGCCACAGAGACAGAATG**	**GCTACAGAGCATGGAAAACAGG**
***Scgn***	**TCTCCTCAAGGGCCTCATTTT**	**TGGATCACCAGGCGATAGG**
***Gucy2c***	**CAGGATCTTTGGGGTGGTTG**	**CCGTGGACTTCAATCTTACTGG**
***Slc29a4***	**TTCTCGCTGCTAATGGGCAT**	**GTGGCTGTTTGAAAGCAGCT**
***Cdhr1***	**AGCTGGACAGAGAAAGGGAAG**	**CGGATGATGTAAGGCTCCTG**
***Celsr2***	**TACATCCCCTTCTTGCTGAGG**	**GATGAGTGGGTGGAGGCATAG**
***Rpl32***	**CAATGTGTCCTCTAAGAACCGAAA**	**CCTGGCGTTGGGATTGG**

### RT-PCR analysis of RNA from mouse tissues

Tissues were obtained from female C57/BL6 mice and immediately homogenized in guanidine isothiocyanate solution. Islets were isolated as previously described [9]. Total RNA extraction and cDNA synthesis were performed as described above. PCR reactions were carried out with Blend Taq (Toyobo) using the following cycling conditions: 94°C for 2 min followed by 25–30 cycles of 94°C for 30 s, 60°C for 30 s, and 72°C for 60 s. *Rpl32* expression was used as an internal control [10,11]. The sequences of the primers used are shown in Table 1.

### Design of short hairpin RNAs (shRNAs)

shRNAs were designed using siDirect 2.0 (http://siDirect2.RNAi.jp/) or the Public TRC Portal website (http://www.broadinstitute.org/rnai/public/seq/search). Five shRNA sequences targeting each candidate gene were selected for evaluation. The shRNA target sequences that were capable of effective knockdown were used in this study and are shown in Table 2. Each of the shRNA oligonucleotides was designed to include the mouse U6 (mU6) promoter sequence upstream of the target sequence (not shown).

**Table 2:** Target sequences of shRNA oligonucleotides used in this study.

Gene	Target sequences #1	Target sequences #2
***Tmem59l***	**CCTCAGAGTCCCCGAATAAC**	
***Scgn***	**GGATAACAGTGTAGAGTTTAT**	**GGATTTGTCAAAGATATGATG**
***Gucy2c***	**GGATGAAGGACCAAGAATACA**	**GTCAACAATGCATCTTTCAAA**
***Slc29a4***	**CCCTTGCTCTTTATCAGCATA**	**CGACTATCTTCACCACAAGTA**
***Cdhr1***	**CCCAGCACTAGAAGTGTCTTT**	**CCCTACTATGGTTACGTGTAT**
***Celsr2***	**GCACAGATCATGTACCAGATT**	**GCTCTGAATTTCTCTTCTTTA**

### Lentiviral vector production and infection of MIN6c4 cells

The lentiviral vector plasmids for shRNA expression were constructed by replacing the CMV-GFP cassette of the SIN vector (CS-CDF-CG-PRE, kindly provided by Dr. H. Miyoshi, RIKEN Tsukuba Institute) with the mU6 promoter-shRNA oligonucleotides described above. The PGK promoter-driven puromycin-resistance gene cassette was used as a selective marker and inserted upstream of the mU6 promoter in the opposite direction. The shRNA-expressing lentiviral vectors were produced by transfecting HEK293T cells with the resulting SIN vector plasmids together with packaging plasmids (pCAG-HIVgp and pCMV-VSV-G-RSV-Rev, kindly provided by Dr. H. Miyoshi), as described previously [12,13]. The lentivirus particles were concentrated with a 4× PEG-it solution [32% (w/v) PEG-6000, 400 mM NaCl, and 40 mM HEPES, pH 7.4]. A negative control viral vector, which lacked an shRNA oligonucleotide insert, was also generated. To produce a lentiviral vector expressing *Tmem59l*, the CMV-GFP cassette of CS-CDF-CG-PRE was replaced with the CAG promoter-driven *Tmem59l* cDNA. The IRES-zeocin-resistance gene cassette was used as a selection marker and inserted downstream of the *Tmem59l* cDNA sequence. Similarly, a lentiviral vector expressing shRNA-resistant *Tmem59l*, which had a silent mutation in the shRNA target site, was also constructed. MIN6c4 cells were seeded in a 12-well plate, cultured overnight, and infected with lentiviral vectors. The infected cells were selected with 1.2 μg/ml puromycin for shRNA expression or 200 μg/ml zeocin for *Tmem59l* expression for 2–3 weeks. The resulting colonies were collected and cultured for use in the experiments described below.

### Measurement of insulin secretion

MIN6c4 knockdown and control cells were cultured in 24-well plates for 2–3 days. Prior to the insulin secretion assay, the cells were starved in Krebs-Ringer bicarbonate buffer (KRBB) containing 10 mM Hepes pH 7.4, 0.2% bovine serum albumin (BSA), and 3 mM glucose for 30 min, and then washed three times with KRBB. The cells were then incubated in KRBB containing Hepes and BSA with 3, 8, or 25 mM glucose, 30 mM KCl plus 3 mM glucose, or 10 nM exendin-4 plus 10 mM glucose for 1 h. The culture medium was collected, and the secreted insulin was measured using an ELISA kit (Mercodia, Uppsala, Sweden). To normalize the amount of secreted insulin, the cells in each well were lysed with RIPA buffer, and the protein concentration of the cell lysates was measured using the Bradford method (Bio-Rad, Hercules, CA, USA). The insulin secretion rate was expressed as the amount of secreted insulin (μg)/mg protein/h [14–16].

### Measurement of insulin content

MIN6c4 knockdown and control cells were cultured in 6-cm dishes for 3–5 days. The cells were trypsinized and washed twice with PBS. The collected cells were then divided into two equal aliquots. One aliquot was extracted with acid-ethanol (0.18 M HCl in 75% ethanol) overnight at -20°C, followed by centrifugation and supernatant collection. The insulin level in the supernatants was determined as indicated above. The other aliquot was lysed with RIPA buffer, and the protein concentration of the extract was determined as described above. The insulin content was expressed as the amount of insulin (μg) per mg protein.

### Generation of an anti-TMEM59L antibody

To raise polyclonal antibodies against TMEM59L, a fusion protein containing glutathione S-transferase (GST) and the N-terminal region of TMEM59L was produced in *E*. *coli*. In brief, a *Tmem59l* cDNA fragment encoding amino acids 1–222 of TMEM59L was amplified by high fidelity PCR using Platinum Pfx DNA polymerase (Invitrogen, Carlsbad, CA, USA). The cDNA fragment was inserted into the pGEX-6P-3 expression vector (GE Healthcare, Buckinghamshire, UK), and the resulting plasmid was introduced into *E*. *coli* to produce the GST-TMEM59L fusion protein, which was purified using a Glutathione Sepharose 4B (GE Healthcare) column. The purified fusion protein was used to generate anti-TMEM59L polyclonal antisera in rabbits. The TMEM59L immunoreactive antiserum was immunoaffinity-purified, and the resulting purified antibody was used as described below.

### Western blotting

MIN6c4 cells were lysed in lysis buffer (20 mM Tris-HCl pH7.5, 150 mM NaCl, and 0.1% NP40) and centrifuged to remove cell debris. SDS-polyacrylamide gel electrophoresis (SDS-PAGE) and Western blotting were carried out using standard techniques. The blots were incubated with either the rabbit anti-TMEM59L antibody (1:1,000) or a mouse anti-α-tubulin antibody (1:50,000, Sigma-Aldrich, Saint Louis, MO, USA), which were detected by horseradish peroxidase (HRP)-conjugated goat anti-rabbit IgG (1:2,000, New England Biolabs, Cambridge, MA, USA) or HRP-conjugated goat anti-mouse IgG1 (1:2,000, Bethyl Lab, Montgomery, TX, USA), respectively. The signals were detected using an enhanced chemiluminescence (ECL) kit (Amersham, Arlington Heights, IL, USA).

### Immunofluorescence analyses

MIN6c4 cells were fixed with 4% paraformaldehyde for 10 min and then permeabilized in PBS containing 0.2% Triton X-100 for 10 min at room temperature. The cells were blocked using Blocking One (Nacalai Tesque, Kyoto, Japan) for 60 min at room temperature followed by incubation with a primary antibody at 4°C overnight and then with a fluorescein-conjugated secondary antibody (1:400) and DAPI (4’, 6-diamidino-2-phenylindole) (Sigma-Aldrich, Saint Louis, MO, USA) for 60 min at room temperature. The stained cells were examined by confocal laser scanning microscopy using a FLUOVIEW FV1000D (Olympus, Tokyo, Japan). The following primary antibodies were used in this analysis: purified rabbit anti-TMEM59L antibody (1:400), guinea pig anti-insulin antibody (1:500, DAKO, Glostrup, Denmark), mouse anti-GM130 antibody (1:500, BD Transduction Laboratories, San Jose, CA, USA), and mouse anti-β-catenin antibody (1:1,000, BD Transduction Laboratories). The following secondary antibodies were used in this analysis: Alexa Fluor 488-labeled goat anti-rabbit IgG, Alexa Fluor 594-labeled goat anti-guinea pig IgG, and Alexa Fluor 647-labeled goat anti-mouse IgG1 (Life Technologies, Grand Island, NY, USA).

### Statistical analysis

The results are presented as the mean ± SD. Statistical analyses were performed using the Student’s t-test. A value of *P*<0.05 was considered statistically significant.

## Results

### mRNA expression analysis

**Fig 1:**
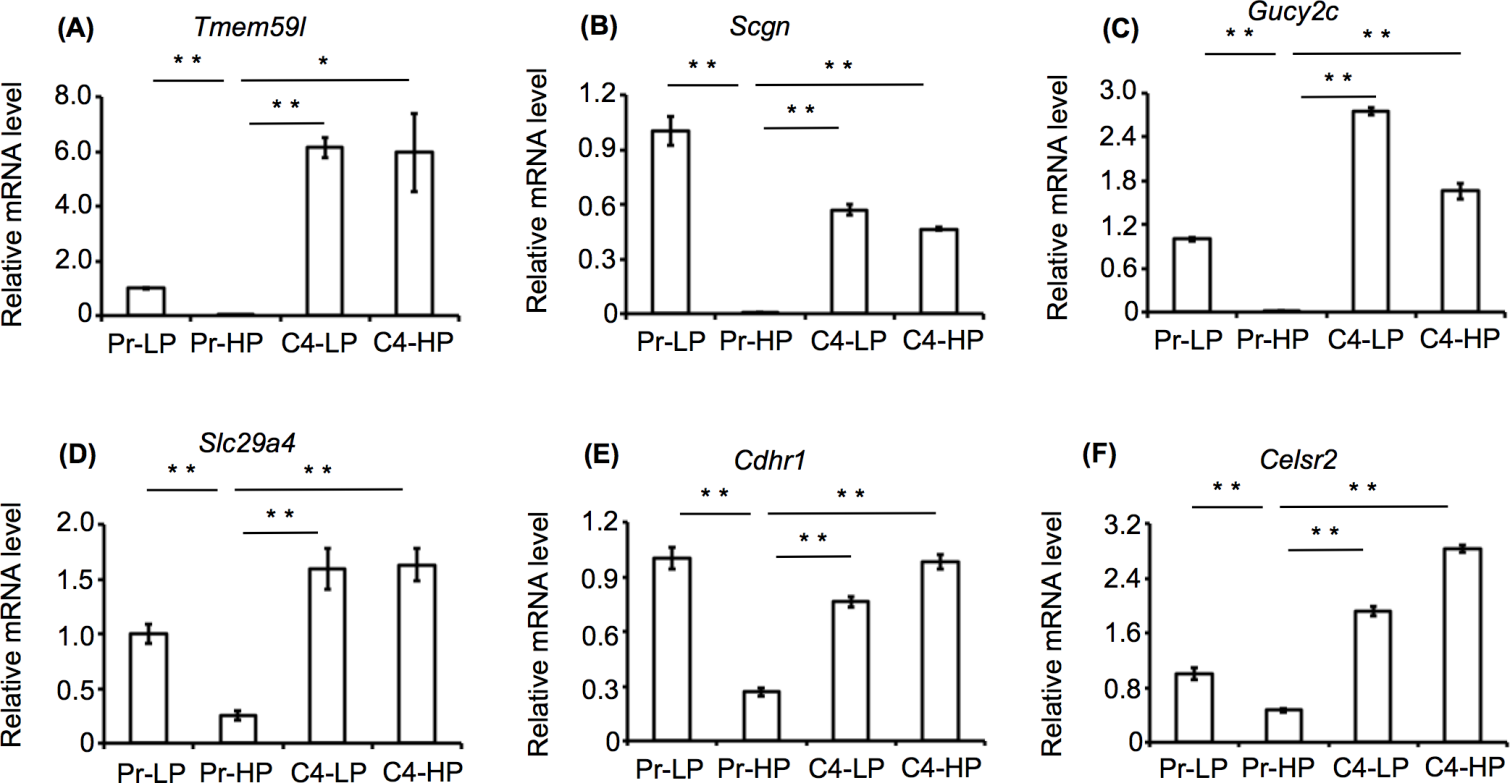
Expression of the selected candidate genes in MIN6 cells. Expression of the *Tmem59l* (A), *Scgn* (B), *Gucy2c* (C), *Slc29a4* (D), *Cdhr1* (E), and *Celsr2* (F) mRNAs in the Pr-LP, Pr-HP, C4-LP, and C4-HP MIN6 cells was examined by quantitative RT-PCR. *Rpl32* gene expression was used as an internal control. Values are the means ± SD (n = 3) of the gene expression levels relative to those in the Pr-LP MIN6 cells. **P<0.005.

The expression patterns of the selected genes, *Tmem59l*, *Scgn*, *Gucy2c*, *Slc29a4*, *Cdhr1*, and *Celsr2* were examined in the Pr-LP, Pr-HP, C4-LP, and C4-HP cells by quantitative RT-PCR. All of these genes were highly expressed in the Pr-LP, C4-LP, and C4-HP cells, but only weakly expressed in the Pr-HP cells (**Fig 1**). These results were consistent with our previously reported findings [4].

**Fig 2:**
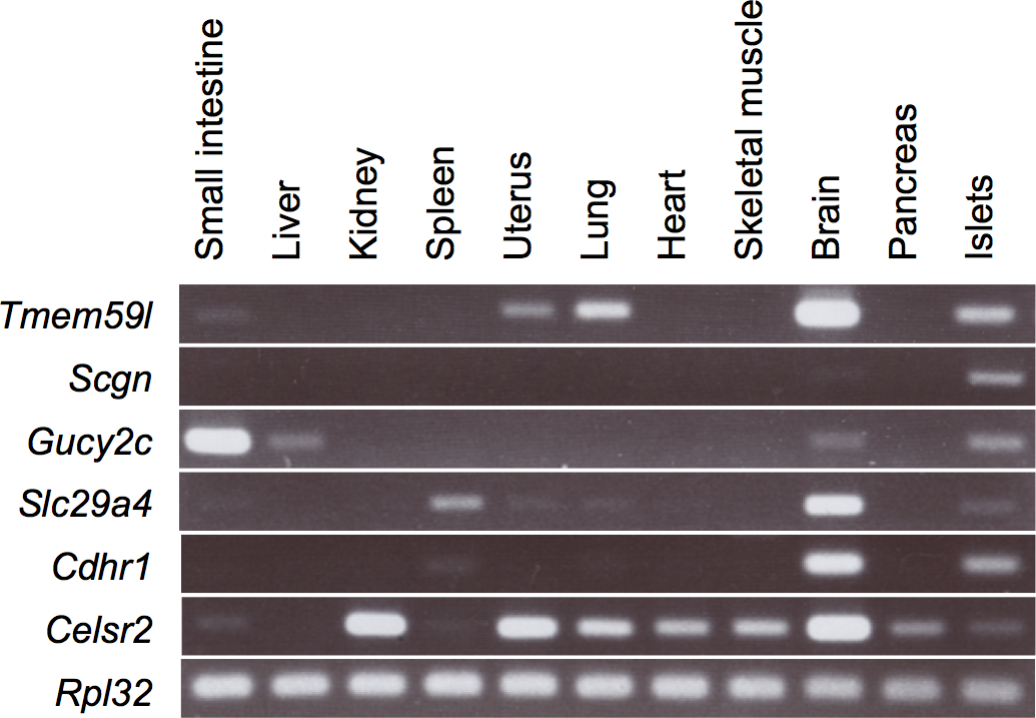
Expression of the selected candidate genes in murine tissues. Expression of the *Tmem59l*, *Scgn*, *Gucy2c*, *Slc29a4*, *Cdhr1*, and *Celsr2* mRNAs in the small intestine, liver, kidney, spleen, uterus, lung, heart, skeletal muscle (quadriceps femoris), brain, pancreas, and islets of mice was examined by RT-PCR. The PCR products were separated by electrophoresis in an agarose gel. *Rpl32* gene expression was used as an internal control.

The expression of these genes in various mouse tissues was examined by RT-PCR (**Fig 2**). Consistent with the databases we referred to (see **Introduction**), *Tmem59l*, *Gucy2c*, *Slc29a4*, and *Cdhr1* were expressed in both the brain and pancreatic islets. In contrast, *Scgn* was expressed in the islets, but not in the brain. The expression of these genes was not detected in the whole pancreas in this experiment, suggesting that their expression was probably restricted to the islets. The expression of *Celsr2* was much broader than that of the other genes, but was only weakly detected in the islets. Thus, most of the examined candidate genes were selectively expressed in the pancreatic islets and brain in mice.

### Effects of selected gene knockdown on GSIS

**Fig 3:**
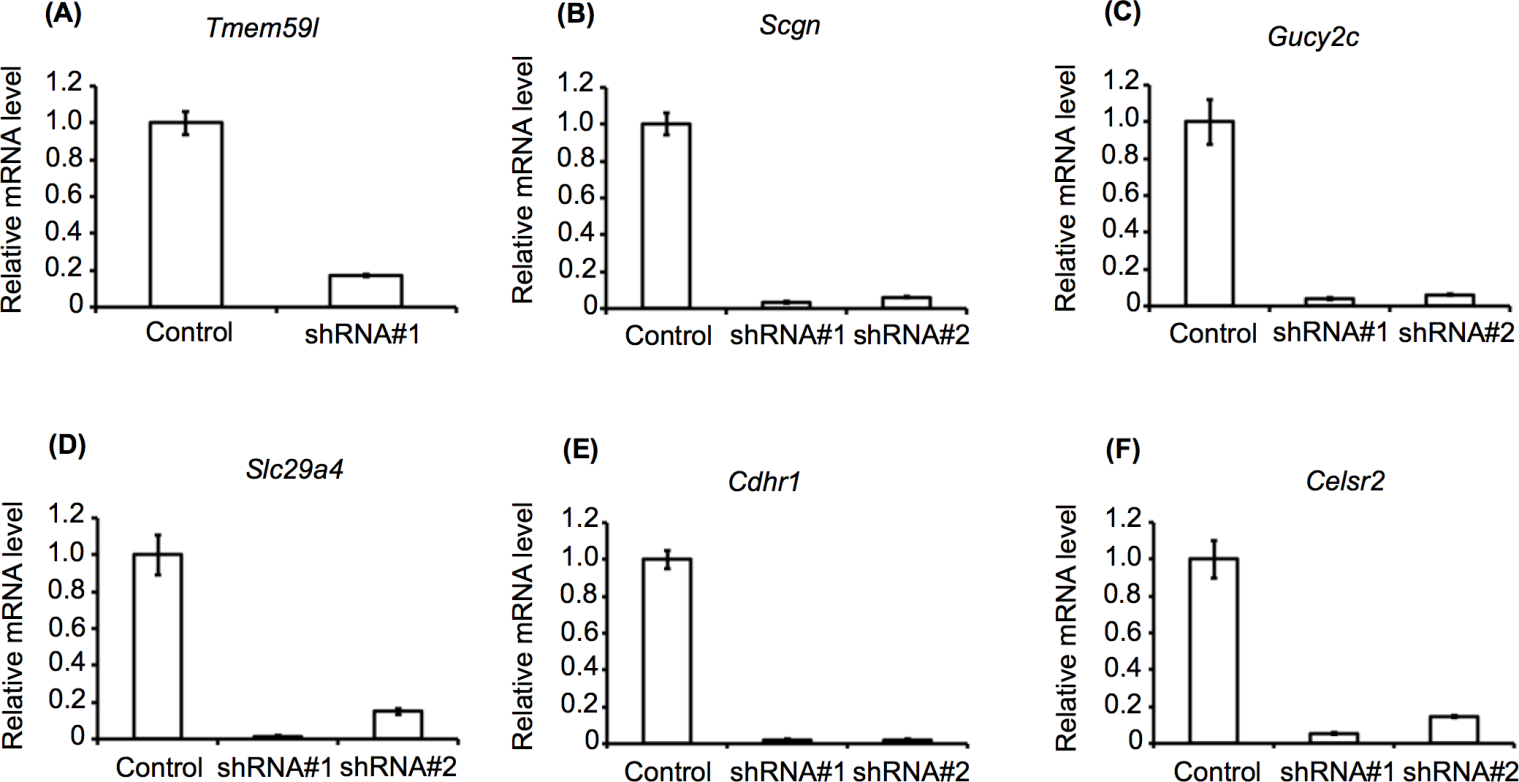
Knockdown of the selected candidate genes in shRNA-expressing MIN6c4 cells. Expression of the *Tmem59l* (A), *Scgn* (B), *Gucy2c* (C), *Slc29a4* (D), *Cdhr1* (E), and *Celsr2* (F) genes in shRNA-expressing MIN6c4 cells was examined by quantitative RT-PCR. *Rpl32* gene expression was used as an internal control. Values are mean ± SD (n = 3) of the gene expression levels relative to those of control MIN6c4 cells that had been infected with an empty lentivirus vector.

**Fig 4:**
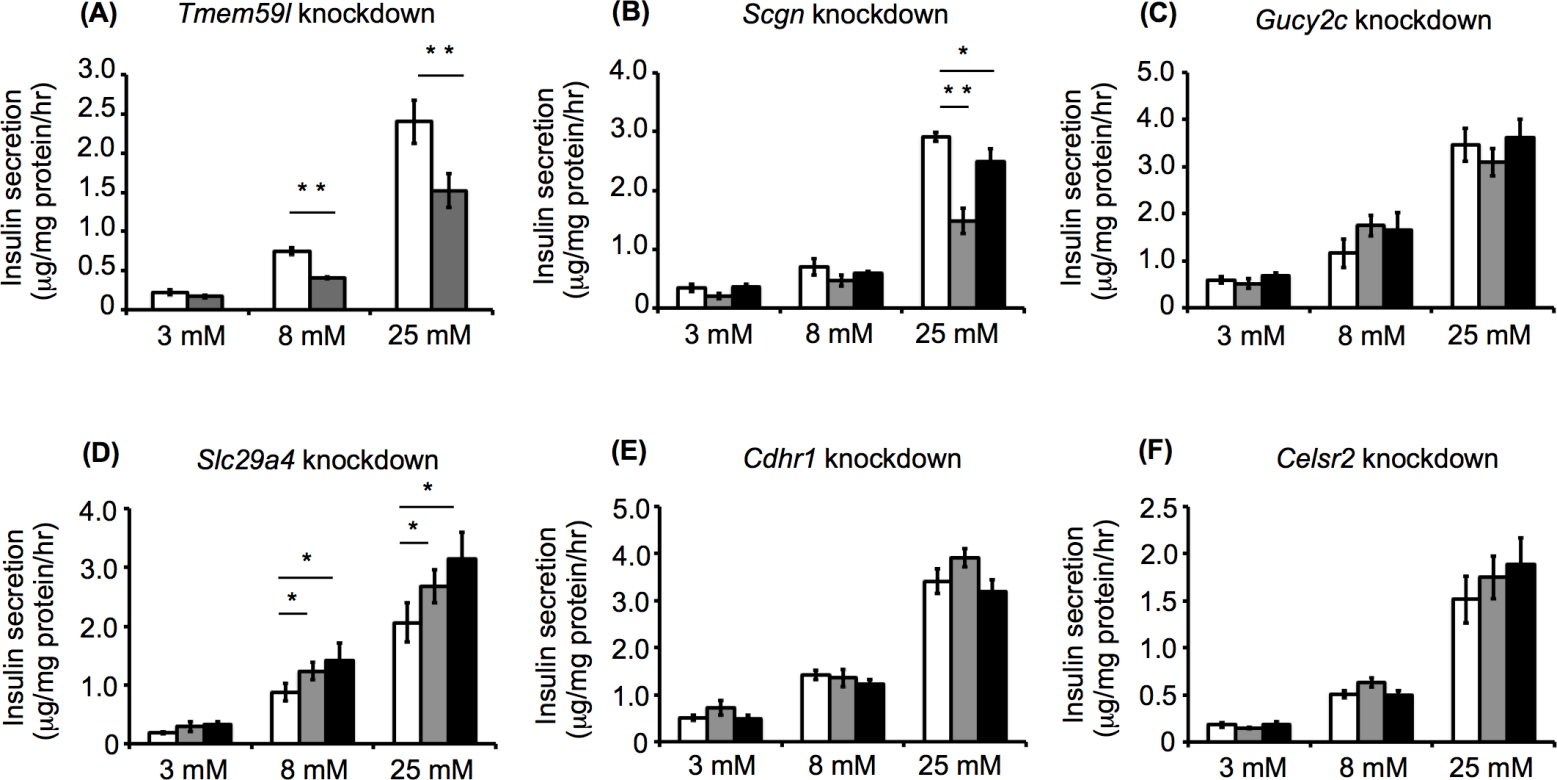
Glucose stimulated insulin secretion from knockdown MIN6c4 cells. Insulin secretion from *Tmem59l* (A), *Scgn* (B), *Gucy2c* (C), *Slc29a4* (D), *Cdhr1* (E), and *Celsr2* (F) knockdown MIN6c4 cells stimulated with 3, 8, or 25 mM glucose (gray bar: shRNA#1, black bar: shRNA#2, and white bar: control). MIN6c4 cells that had been infected with an empty lentivirus vector were used as the control. Values are means ± SD (n = 3–4). ^*^P<0.05 ^**^P<0.005.

Knockdown of the selected genes in MIN6c4 cells was performed by the lentivirus-mediated expression of specific shRNAs. Quantitative RT-PCR was used to evaluate the effectiveness of the shRNAs, and led to the identification of one or two shRNAs that effectively knocked down each gene in the MIN6c4 cells (**Fig 3**). The analysis of GSIS in the knockdown cells showed that the *Tmem59l* and *Scgn* knockdown cells exhibited reduced GSIS, whereas the *Slc29a4* knockdown cells exhibited enhanced GSIS, compared with the control cells (**Fig 4**). These data suggested that *Tmem59l*, *Scgn*, and *Slc29a4* are functionally related to GSIS in MIN6c4 cells. In contrast, the *Gucy2c*, *Cdhr1*, and *Celsr2* knockdowns did not show any significant effects on GSIS.

### Effects of selected gene knockdown on KCl-stimulated insulin secretion

**Fig 5:**
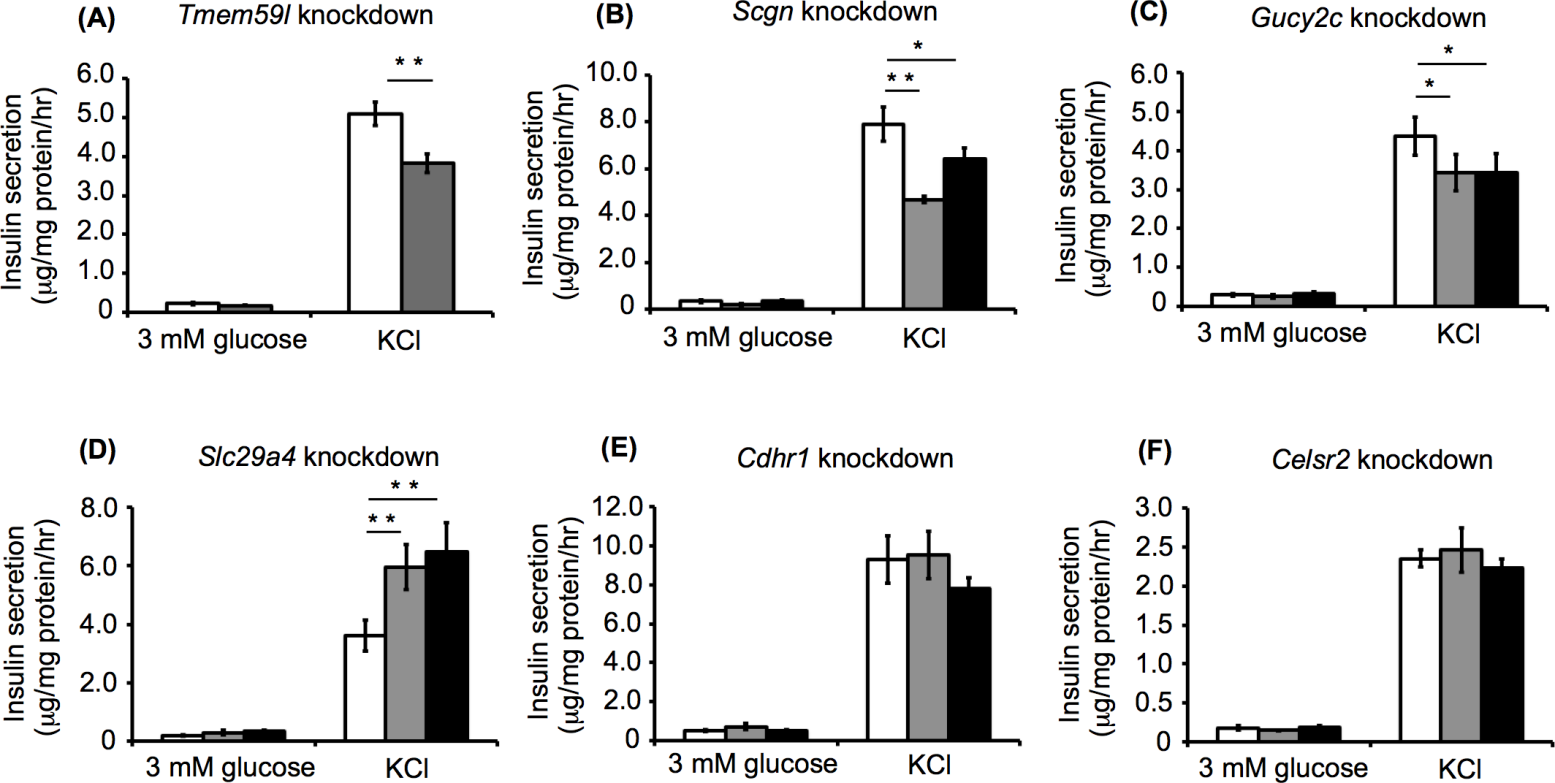
KCl-stimulated insulin secretion from knockdown MIN6c4 cells. Insulin secretion from *Tmem59l* (A), *Scgn* (B), *Gucy2c* (C), *Slc29a4* (D), *Cdhr1* (E), and *Celsr2* (F) knockdown MIN6c4 cells stimulated with 30 mM KCl (gray bar, shRNA#1; black bar, shRNA#2; and white bar, control). Values are means ± SD (n = 3–4). ^*^P<0.05 ^**^P<0.005.

High concentrations of KCl induce β cell plasma membrane depolarization and can more potently stimulate insulin secretion than high concentrations of glucose. Thus, KCl has been used to evaluate the final process following membrane depolarization in the regulated insulin secretory pathway [17–19]. We examined insulin secretion in response to KCl using the knockdown cells. Similar to the results obtained for GSIS, the *Tmem59l* and *Scgn* knockdown cells exhibited reduced insulin secretion, whereas *Slc29a4* knockdown cells showed enhanced insulin secretion compared with the control cells. *Cdhr1* and *Celsr2* knockdown cells showed no significant difference in insulin secretion compared with the control cells. However, while *Gucy2c* knockdown had no effect on GSIS, the *Gucy2c* knockdown cells exhibited lower insulin secretion than the control cells when stimulated with KCl (**Fig 5**). These results provided further evidence that *Tmem59l*, *Scgn*, and *Slc29a4* are functionally involved in the insulin secretory mechanism, and suggested that *Gucy2c* is functionally involved in insulin secretion after β cell membrane depolarization.

### Effects of selected gene knockdown on cellular insulin content

**Fig 6:**
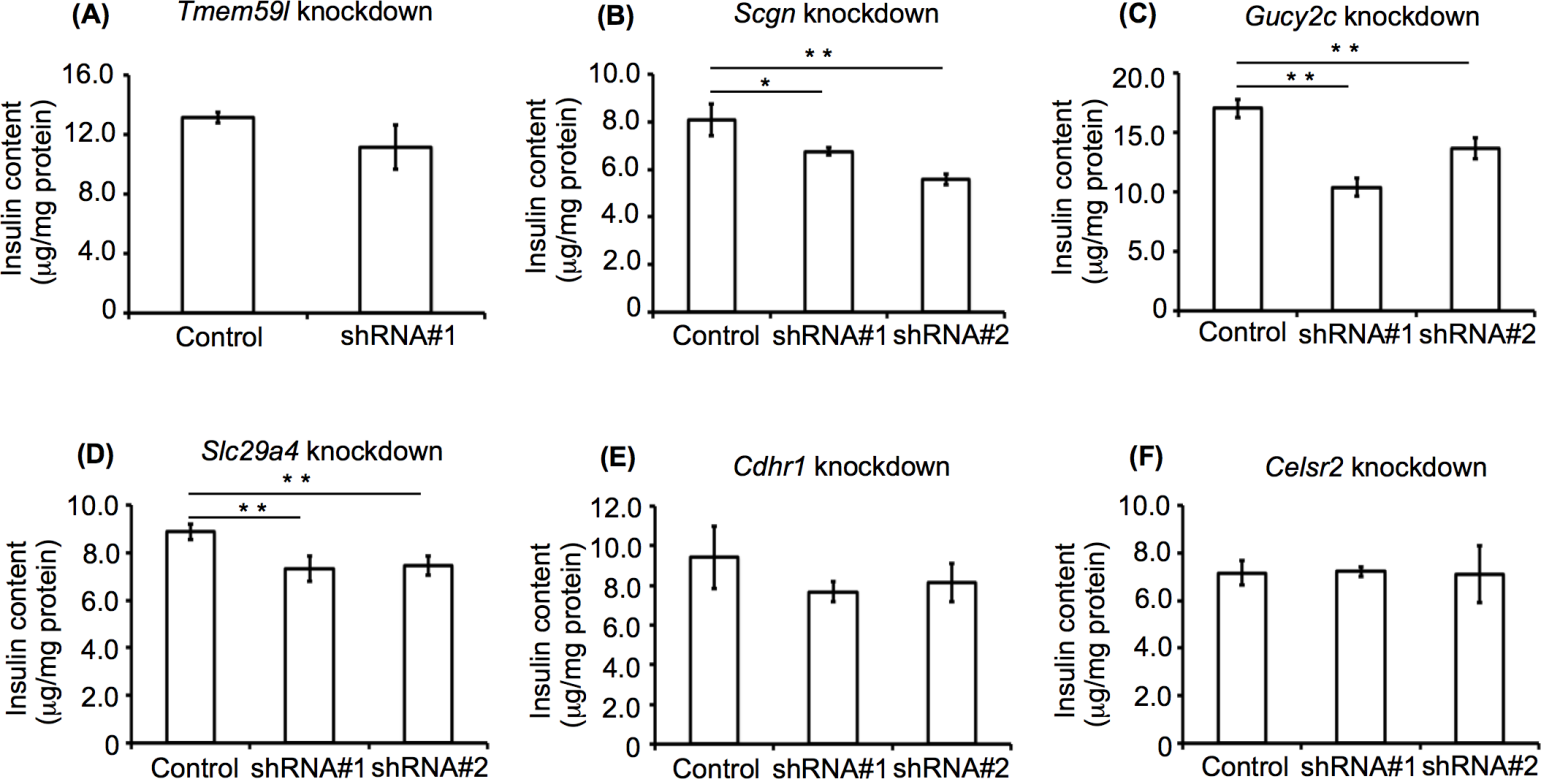
Insulin content of knockdown MIN6c4 cells. Insulin content of *Tmem 59l* (A), *Scgn* (B), *Gucy2c* (C), *Slc29a4* (D), *Cdhr1* (E), and *Celsr2* (F) knockdown MIN6c4 cells, compared to control cells containing empty lentiviral vectors. Values are means ± SD (n = 3–4). ^*^P<0.05 ^**^P<0.005.

We next examined the insulin content of the knockdown cells (**Fig 6**). *Scgn*, *Gucy2c*, and *Slc29a4* knockdown cells exhibited lower cellular insulin levels than the control cells. In contrast, the *Tmem59l*, *Cdhr1*, and *Celsr2* knockdown cells showed no significant differences in insulin content compared with the control cells. These data suggested that *Scgn*, *Gucy2c*, and *Slc29a4* may be functionally involved in regulating cellular insulin levels as well as insulin secretion, while *Tmem59l* may be specifically involved in regulating insulin secretion.

### Functional analysis of *Tmem59l*

**Fig 7:**
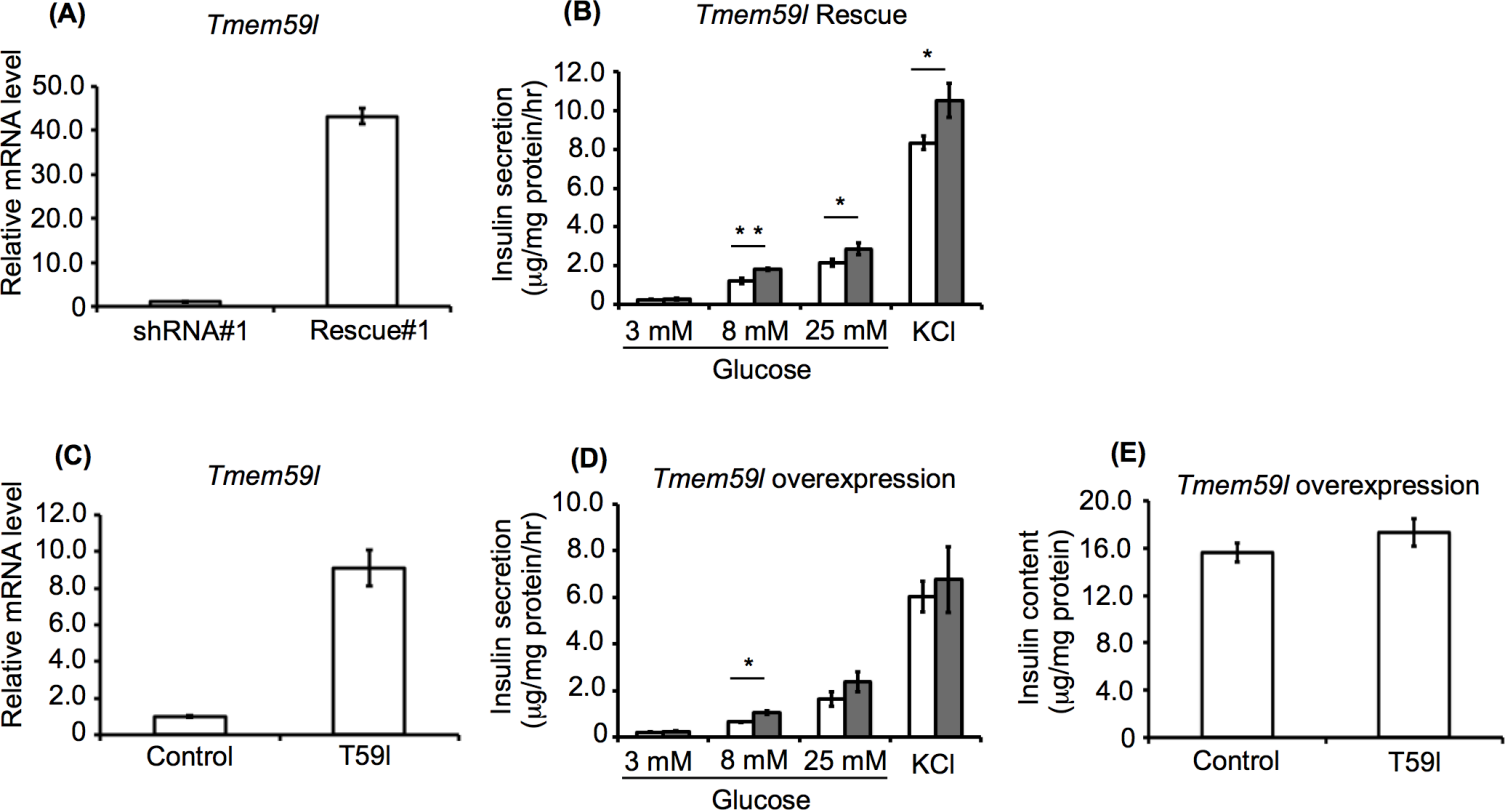
Insulin secretion and content of *Tmem59l* rescued and overexpressing MIN6c4 cells. (A) Expression of the *Tmem59l* gene in *Tmem59l* rescued (Rescue#1) MIN6c4 cells. (B) Insulin secretion from Rescue#1 MIN6c4 cells stimulated with 3, 8, and 25 mM glucose or 30 mM KCl (white bar: shRNA#1 cells and gray bar: Rescue#1 cells). (C) *Tmem59l* gene expression in the *Tmem59l*-overexpressing (T59l) MIN6c4 cells. (D) Insulin secretion from T59l MIN6c4 cells stimulated with 3, 8, and 25 mM glucose or 30 mM KCl (white bar, control MIN6c4 cells and gray bar, T591 cells). (E) Insulin content of the T59l cells compared to the control MIN6c4 cells. Values are means ± SD (n = 3–4). ^*^P<0.05 ^**^P<0.005.

The initial analysis of the five *Tmem591*-specific shRNA sequences indicated that only one was capable of efficiently downregulating *Tmem591* expression. Thus, we could not rule out the possibility that the effects of the *Tmem59l* shRNA we observed were due to non-specific gene silencing, or off-target effects. To confirm that the decrease in insulin secretion was due to *Tmem59l* knockdown, we expressed a shRNA-resistant form of *Tmem59l* in the knockdown cells and evaluated its effect on insulin secretion in these cells. The resulting *Tmem59l* rescued cells exhibited higher levels of secreted insulin than the knockdown cells when stimulated with high concentrations of either glucose or KCl (**Fig 7A and 7B**). These results demonstrated that the reduced insulin secretion observed in the *Tmem59l* knockdown cells was due to the specific knockdown of *Tmem59l* mRNA. Moreover, *Tmem59l*-overexpressing MIN6c4 cells showed a tendency to secrete higher levels of insulin compared with the parental MIN6c4 cells. However, the insulin content was not significantly increased in the *Tmem59l*-overexpressing cells (**Fig 7C–7E**). These results provided further evidence that *Tmem59l* is functionally involved in regulating the insulin secretion from MIN6c4 cells.

**Fig 8:**
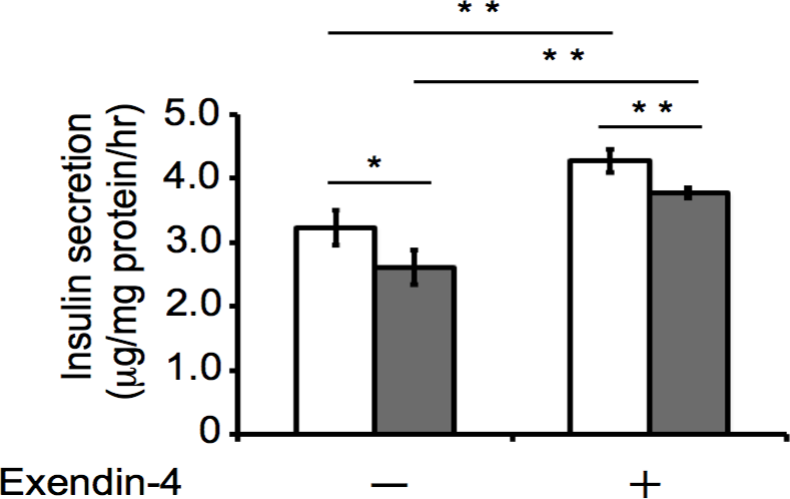
Effect of exendin-4 on GSIS from *Tmem59l* knockdown cells. Insulin secretion from the *Tmem59l* knockdown (gray bar: shRNA#1) and control (white bar) MIN6c4 cells stimulated with 10 mM glucose in the absence or presence of 10 nM exendin-4. Values are means ± SD (n = 3–4). ^*^P<0.05 ^**^P<0.005.

We also examined whether *Tmem59l* knockdown affects the potentiation of GSIS by exendin-4, a GLP-1 analogue. Although exendin-4 potentiated insulin secretion stimulated with 10 mM glucose in both *Tmem59l* knockdown and control MIN6c4 cells, the knockdown cells still exhibited reduced insulin secretion compared with the control cells (**Fig 8**). This result suggested that *Tmem59l* is not directly involved in the potentiation of GSIS by GLP-1.

### TMEM59L protein localization in MIN6c4 cells

**Fig 9:**
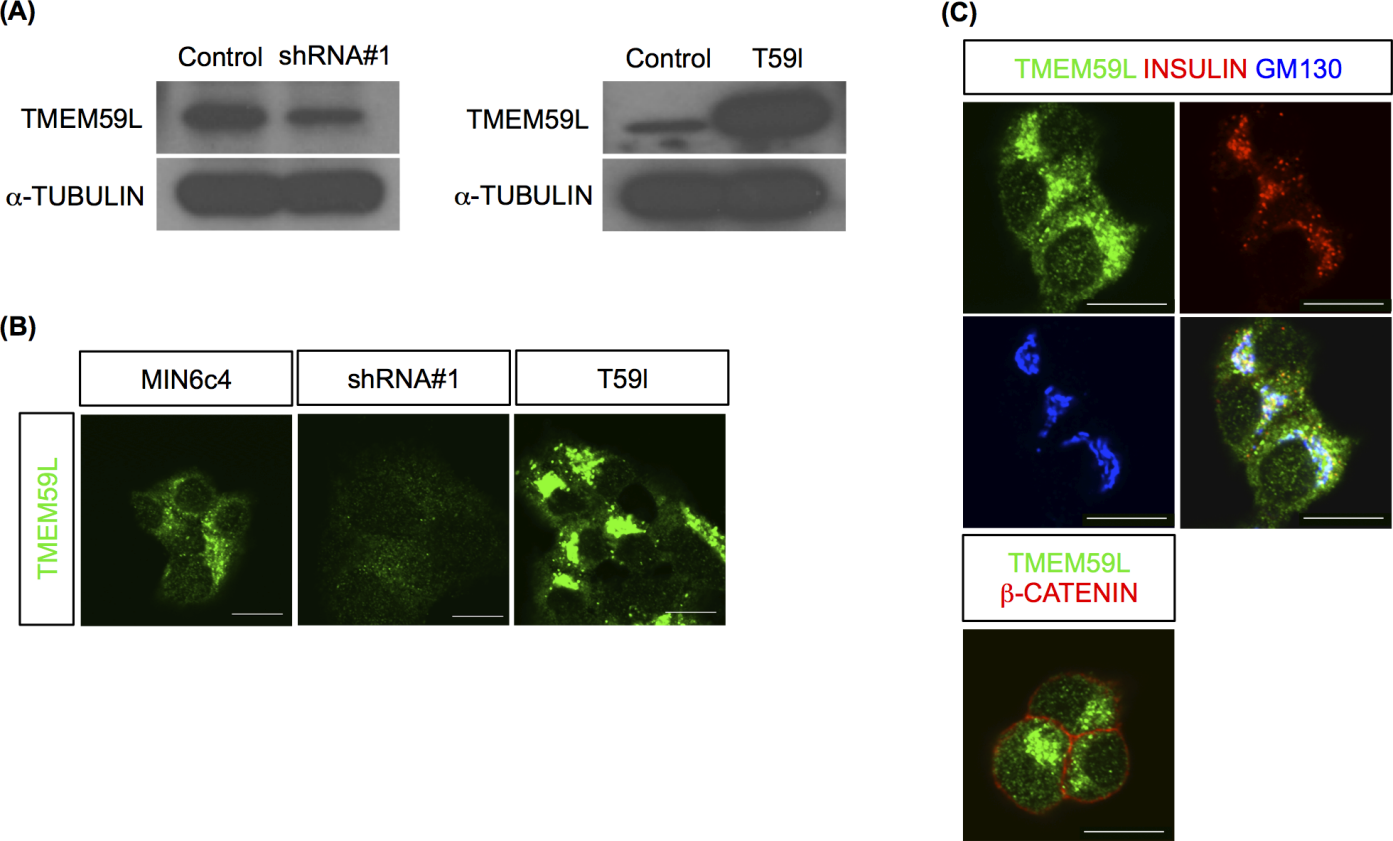
Localization of the TMEM59L protein in MIN6c4 cells. (A) Detection of the TMEM59L protein in control, *Tmem59l* knockdown (shRNA#1), and *Tmem59l*-overexpressing (T59l) MIN6c4 cells by Western blotting with the anti-TMEM59L antibody, using α-tubulin as a loading control (B) Immunofluorescence staining of control, shRNA#1, and T59l MIN6c4 cells using the anti-TMEM59L antibody. (C) Localization of the TMEM59L protein in MIN6c4 cells. Antibodies against GM130 and β-catenin stained the Golgi complex and plasma membrane, respectively. Scale bars: 10 μm.

Next, we generated an anti-TMEM59L antibody (see **Materials and Methods**) to examine the localization of TMEM59L protein in MIN6c4 cells. We demonstrated its specificity for detecting TMEM59L protein (predicted mass: 43 kDa) by Western blotting using *Tmem59l* knockdown and overexpressing MIN6c4 cells (**Fig 9A**). Furthermore, we compared the signal intensity of immunofluorescence staining with the anti-TMEM59L antibody among *Tmem59l* knockdown, overexpressing, and control MIN6c4 cells. The result clearly showed that the signal intensity was decreased by *Tmem59l* knockdown, while greatly increased by *Tmem59l* overexpression **(Fig 9B)**. Immunofluorescence analysis of MIN6c4 cells with this antibody showed that TMEM59L colocalized with insulin and with the GM130 Golgi complex marker. In contrast, TMEM59L did not colocalize with β-catenin, a plasma membrane marker (**Fig 9C**). These results suggested that TMEM59L localizes to insulin granules and the Golgi complex and may play a role in insulin secretion.

## Discussion

We previously identified several genes that may be functionally involved in maintaining insulin secretion in MIN6c4 β cells [4]. In the present study, we used shRNA knockdown to study the functional involvement of some of these genes with insulin secretion and content in MIN6c4 cells. The knockdown of *Scgn* and *Gucy2c* resulted in decreased insulin secretion and content, while the knockdown of *Tmem59L* resulted in reduced insulin secretion, without affecting insulin content. In contrast, *Slc29a4* knockdown resulted in increased insulin secretion and decreased cellular insulin levels. The finding that *Slc29a4* knockdown resulted in increased insulin secretion was unexpected, because the selected genes were upregulated in MIN6c4 cells, a MIN6 subclone that maintains insulin secretion after long-term culture [4]. On the other hand, *Cdhr1* and *Celsr2* knockdown did not affect the insulin secretion or insulin content (**Figs 4–6**).

The *Scgn* cDNA was first cloned from a pancreatic β cell cDNA library and encodes a hexa EF-hand calcium-binding protein [20]. *Scgn* overexpression in MIN6 cells was previously shown to enhance GSIS and it was proposed that SCGN might interact with SNAP-25 to enhance GSIS as a Ca^2+^-signaling protein [21–23]. These reports are consistent with our results, but the reason for the decreased insulin content seen in the *Scgn* knockdown MIN6c4 cells is not clear. *Gucy2c* knockdown had no significant effect on GSIS, but decreased KCl-induced insulin secretion and cellular insulin content. KCl is a more potent stimulator of insulin secretion than high glucose, so that insulin secretion stimulated with KCl is likely to be more susceptible to the alternations in insulin content than that with high glucose. Therefore, it might be possible that the reduced insulin content led to a decrease in KCl-induced insulin secretion. However, the reason why *Gucy2c* knockdown decreased the insulin content in MIN6c4 cells is not clear. *Gucy2C* was reported to be expressed in the small intestine and encodes one of seven mammalian transmembrane guanylate cyclase receptors that catalyze the formation of cGMP in response to the binding of the bacterial heat-stable enterotoxin STa or of the endogenous peptides guanylin and uroguanylin [24–26]. Although the function of the *Gucy2c*-encoded guanylate cyclase receptor in pancreatic β cells has not been reported, guanylin has been shown to stimulate insulin secretion in a rat pancreatic β cell line [27], suggesting a potential role for its receptor in insulin secretion.

*Slc29a4* knockdown enhanced insulin secretory response to glucose and KCl, while it reduced cellular insulin content. It appears that increased insulin secretion caused a decrease in insulin content. In fact, Bollheimer et al. [28] showed that exposure of rat islets to free fatty acid enhanced basal and glucose-stimulated insulin secretion and reduced insulin content. Eto et al. [29] investigated the β-cell function of phosphatidylinositol 3-kinase p85α regulatory subunit-deficient mice and showed that p85α knockout enhanced GSIS from isolated islets and reduced both insulin content and insulin secretion stimulated with diazoxide and KCl. However, in these two reports, the decrease in insulin content was attributed to lowered insulin biosynthesis. Thus, the cause-and-effect relationship between insulin content and insulin secretory capacity seems complicated. Further analysis will be required to understand the role of *Slc29a4* in the regulation of insulin content and insulin secretion. *Slc29a4* was identified as one of a family of equilibrative nucleoside transporter (ENT) genes. Its gene product transports not only nucleosides but also monoamines (e.g., serotonin) [30,31]. Although *Slc29a4* has not been directly linked to insulin secretion, nucleosides and monoamines were shown to regulate insulin secretion in pancreatic β cells [32–36], consistent with a potential role for this solute carrier in insulin secretion.

*Cdhr1* was identified as one of three non-classical cadherin genes [37] and was reported to be a candidate gene for retinal dystrophies [38]. *Celsr2* was identified as a mammalian homolog of the *Drosophila* gene *flamingo*, which encodes a receptor involved in noncanonical Wnt signaling [39]. Mouse *Celsr2* was reported to control the differentiation of pancreatic β cells from polarized progenitors [40]. In the present study, the downreglation of these genes had no significant effect on insulin secretion or cellular insulin content; however we cannot rule out the possibility that the low levels of *Cdhr1* and *Celsr2* expressed in the knockdown cells were sufficient for maintaining their functions.

*Tmem59l* knockdown resulted in decreases in both glucose- and KCl-stimulated insulin secretion, but did not significantly alter the cellular insulin content (**Figs 4–6**). Considering that the *Tmem59l* knockdown cells still expressed considerable levels of *Tmem59l* mRNA (**Fig 3**), the *Tmem59l* gene product may play an essential role in maintaining insulin secretion. We confirmed the specificity of the *Tmem59l* knockdown by showing that *Tmem59l* overexpression could rescue the phenotype of the *Tmem59l* knockdown cells (**Fig 7A and 7B**). Moreover, we found that *Tmem59l*-overexpressing cells tended to exhibit enhanced insulin secretion compared with the control cells (**Fig 7C–7E**). These results were consistent with the notion that the *Tmem59l* gene product positively regulates insulin secretion.

The human *TMEM59L* gene is also known as *BSMAP* (brain-specific membrane-anchored protein). *BSMAP* was identified as a gene that is highly expressed in the brain and is localized to chromosome 19p12 [41]. The predicted structure of the TMEM59L protein suggests that it is a membrane-bound type 1 glycoprotein. TMEM59L was previously shown to be involved in peripheral axon extension in sensory neurons [42]. Recently, Ullrich et al. [43] identified TMEM59, a homolog of TMEM59L, as a modulator of amyloid precursor protein (APP) shedding. TMEM59 was found to be a ubiquitously expressed, Golgi-localized protein. Notably, TMEM59L was shown to have a similar inhibitory effect on APP maturation and shedding as TMEM59, suggesting that both proteins have similar functions in the brain [43]. However, the function and subcellular localization of TMEM59L in pancreatic β cells have not been reported previously. We examined the localization of TMEM59L protein in MIN6c4 cells by immunofluorescence staining and found that TMEM59L preferentially colocalized with a Golgi complex marker. We also found that TMEM59L protein colocalized with insulin granules (**Fig 9C**), which are known to be generated from the Golgi complex (reviewed in [44]). On the other hand, colocalization of TMEM59L with markers for mitochondria, endoplasmic reticulum (ER), or nuclei was not observed (**S1 Fig**). Further studies will be needed to exactly determine the localization of TMEM59L in β cells.

Our present study suggested that several of the candidate genes identified in our previous microarray analysis, which compared gene expression in MIN6c4 cells and the parental MIN6 cells and the MIN6c4 cells, are functionally involved in GSIS. We focused on *Tmem59l* and found that the TMEM59L protein localized to insulin granules and the Golgi complex, suggesting that it might play important roles in insulin transport and secretion. We expect that further analyses of these candidate genes will contribute to the elucidation of the insulin secretory pathway.

## Supporting Information

S1 Fig Co-staining of TMEM59L with mitochondria, endoplasmic reticulum (ER), and nucleus markers in MIN6c4 cells. Green: staining with anti-TMEM59l antibody; red: staining with MitoTracker Red CMXRos (Life Technologies), used as a mitochondria marker; blue: staining with ER-Tracker Blue-White DPX (Life Technologies), used as an ER marker; light blue: staining with DAPI, used as a nucleus marker.
(TIFF)
