# Supplementary figures and images for: Functional Analysis of Novel Candidate Regulators of Insulin Secretion in the MIN6 Mouse Pancreatic β Cell Line
Source: PLoS One. 2016 Mar 17;11(3):e0151927. doi: 10.1371/journal.pone.0151927 (PMC4795703; doi:10.1371/journal.pone.0151927)

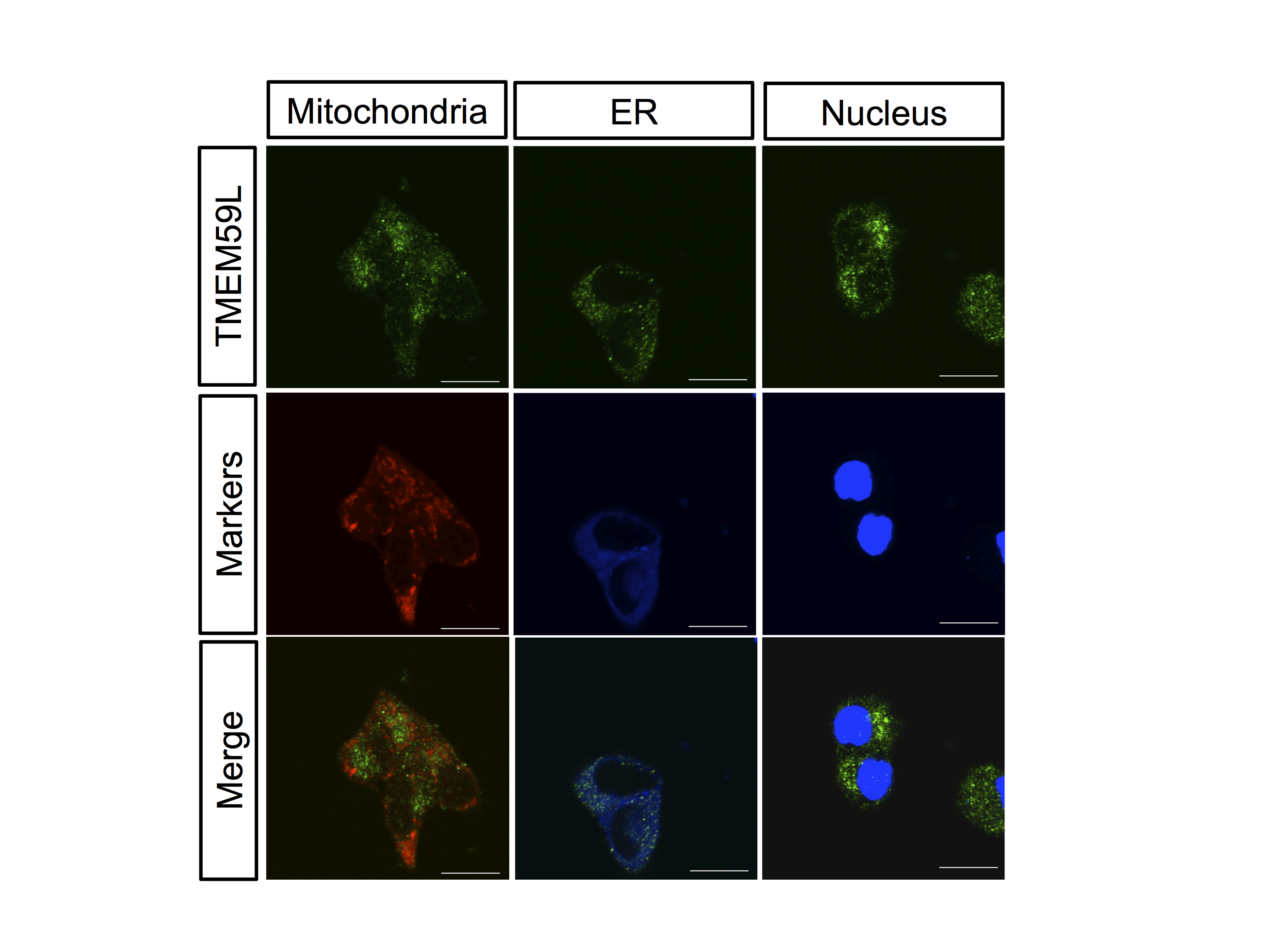

Supplement: S1 Fig — Green: staining with anti-TMEM59l antibody; red: staining with MitoTracker Red CMXRos (Life Technologies), used as a mitochondria marker; blue: staining with ER-Tracker Blue-White DPX (Life Technologies), used as an ER marker; light blue: staining with DAPI, used as a nucleus marker. (TIFF) [file pone.0151927.s001.tiff]
